# The Association of Perceived Neighbourhood Environment and Subjective Wellbeing in Migrant Older Adults: A Cross-Sectional Study Using Canonical Correlation Analysis

**DOI:** 10.3390/ijerph20054021

**Published:** 2023-02-23

**Authors:** Yuxi Liu, Huanting Liu, Qin Chen, Junhui Xiao, Chonghua Wan

**Affiliations:** 1 School of Humanities and Management, Guangdong Medical University, Dongguan 523808, China; 2 Institute of Health Law and Policy, Guangdong Medical University, Dongguan 523808, China; 3 Research Center for Quality of Life and Applied Psychology, Guangdong Medical University, Dongguan 523808, China

**Keywords:** subjective wellbeing, perceived neighbourhood environment, migrant older adults

## Abstract

Existing studies often focus on the impact of the neighbourhood environment on the subjective wellbeing (SWB) of the residents. Very few studies explore the impacts of the neighbourhood environment on migrant older adults. This study was conducted to investigate the correlations between perceived neighbourhood environment (PNE) and SWB among migrant older adults. A cross-sectional design was adopted. Data were collected from 470 migrant older adults in Dongguan, China. General characteristics, levels of SWB, and PNE were collected via a self-reported questionnaire. Canonical correlation analysis was performed to evaluate the relationship between PNE and SWB. These variables accounted for 44.1% and 53.0% of the variance, respectively. Neighbourhood relations, neighbourhood trust, and similar values in social cohesion made the most important contributions correlated with positive emotion and positive experience. A link between SWB and walkable neighbourhoods characterized by opportunities and facilities for physical activities with other people walking or exercising in their community, is positively associated with positive emotions. Our findings suggest that migrant older adults have a good walkable environment and social cohesion in neighbourhoods positively correlated with their subjective wellbeing. Therefore, the government should provide a more robust activity space for neighbourhoods and build an inclusive community for older adults.

## 1. Introduction

In recent years, people have paid an increasing amount of attention to individual happiness aside from economic utility and tend to use subjective wellbeing (SWB) to evaluate social progress [1,2]. SWB refers to an individual’s subjective feelings about his or her life, or the overall feeling of happiness in life [3]. Research on SWB is attentive to people’s values, emotions and evaluation, but does not fully recognise the external judgement of behavioural experts [4]. With regards to the study of SWB, early scholars predominantly focused on the impact of individual ‘endogenous characteristics’ on SWB, primarily referring to socioeconomic attributes such as age, gender, education, family income, marital status, and health status [5,6]. Empirical studies have demonstrated that demographic factors can only explain part of the difference in SWB, whilst some scholars began to pay attention to the influence of ‘exogenous factors’, such as social and residential environment on SWB [7,8,9]. There are relatively numerous studies on the influence of social factors on SWB, and they focus on the influence of social support on SWB [10,11,12]. Existing studies have demonstrated that subjective support, objective support, and support utilisation have moderate positive correlations with overall SWB, life satisfaction, and positive emotions, and moderate negative correlations with negative emotions [10,11,12]. There are some studies exploring SWB from the perspective of neighbourhoods. Hooghe and Vanhoutte’s (2011) research discovered that neighbourhoods with a strong homogeneity have a weaker impact on SWB than neighbourhoods with strong heterogeneity [13]. Research conducted by Chen and Ning (2015) revealed that good neighbourhood relationships, frequent participation in activities, convenient shopping, and beautiful landscapes are the primary neighbourhood environmental factors that affect residents’ SWB [14].

As a basic daily living space for urban residents, it is necessary to weigh the factors of the neighbourhood environment that directly affect people’s lives and feelings about it. Existing studies have demonstrated that the neighbourhood influences people’s SWB, however, the factors may be different at various stages of life [15,16]. The effects of the neighbourhood environment are less important in early to middle adulthood since they work and play outside the neighbourhood more often than older adults [16,17]. Compared to young people, older adults have different behaviours, mobility, and perceptions, and the demand for services and facilities can be extremely different [18,19]. These factors may influence and lead to differences in neighbourhood environmental needs and preferences at different stages of life. For numerous older adults, the neighbourhood in which they live is their primary environmental context [20]. The physical and social conditions of the neighbourhood environment may be more important to older adults, especially those who are retired or becoming frail. Thus, they may spend an increasing amount of time with neighbours in their neighbourhood [9,20].

In addition, existing studies often focus on the impact of the neighbourhood environment on SWB for the residents. However, for most people, the neighbour environment is not fixed, especially in recent years when residential migration has become increasingly common [21,22]. As an overall assessment of a person’s long-term quality of life [23], SWB is necessarily linked to life choices such as migration. Studies have demonstrated that the relocation of residence may cause changes in the living environment, which in turn have an impact on SWB [24,25]. From the perspective of spatial dimension, the longer the distance, the more the migrant may experience greater life changes. These changes are not only related to the support degree of the original social network and capital but also related to the challenge of adapting to the new environment [26,27,28]. Some studies believe that residential migration can lead to significant changes in specific areas of life, bringing about changes in life satisfaction [29,30,31]. Residential migration is often accompanied by specific life course events. However, the environmental changes conveyed by migration at different life course stages and the potentially important role of adaptation to the new environment in the relationship between the neighbourhood environment and SWB have received little attention.

Due to China’s urbanisation, a large number of workers and their families from across the country have migrated to work and live in new places, especially those with high economic status, such as Dongguan and Guangzhou, where jobs are more plentiful and lucrative [11,22]. The ‘one-child’ policy has reduced the size of Chinese families to a two parents and one-child ratio. Corresponding with filial piety as a major Chinese traditional value, Chinese older adults’ family members are brought along with their children who migrate for work to new places with the responsibility to take care of their parents. Other reasons for older adults’ migration include taking care of their grandchildren and reuniting with their families [22,32]. Internal migrant older adults in China, who are accompanied by their adult child migrate to the new place, viewed as ‘floating older adults’ or ‘senior drifters’ [5,32]. The number of migrant older adults has grown rapidly due to the persistence of internal migration and ageing trends in China [5,22]. The existing research on migrant older adults has gradually shifted from focusing on the migration patterns to the effects and causes of these people to the quality of life of migrant older adults [33]. For example, taking care of grandchildren within the family will significantly increase the life satisfaction of migrant older adults which has been reported in some studies [5,22]. The social support of the government and the community has a significant positive impact on the social integration of migrant older adults [34]. However, most of the existing research focuses on the fields of sociology and psychology, focusing on the impact of the social environment on the SWB or quality of life of this group, and few studies consider the impact of the neighbourhood environment [8,14]. Social cohesion is a social neighbourhood factor that affects SWB and is particularly relevant to older adults since it is associated with neighbourhood social order and violent crime rates [20]. In fact, after these elderlies migrated to the city, most of their outdoor and social activities were limited to the neighbourhood, which became the most important social support space for these migrant older adults [8]. The range of functional mobility and communication in cities for migrant older adults is much lower than that of local residents, and they tend to spend most of their time close to home [8,9,35]. Therefore, the study of the SWB of migrant older adults should take into account neighbourhood environment factors. Compared to objective indicators of the neighbourhood environment, the present study believes that the relationship between the subjective perception of the neighbourhood environment and SWB is more direct. In explaining SWB, subjectively perceived features of the neighbourhood environment are often more statistically significant than objective descriptions of environmental elements [36].

The attributes of the neighbourhood environment, and their relationship to SWB, are relatively well researched in Western countries [37,38], however, remain largely underexplored in China. Regarding the impact of the neighbourhood environment on SWB, most of the existing studies believe that positive features of the neighbourhood environment (e.g., walkability, availability of public services, and amenities) are associated with positive SWB [11,14], and most of these studies focus on the role of accessibility. For example, good accessibility to parks and green spaces can provide residents with open and natural public spaces, which has a positive impact on SWB [39]. Yet, few studies have explored the relationship between neighbourhood environment and SWB in Chinese migrant older adults. Even fewer studies examine the association of perceived neighbourhood environment (combined physical and social environment attributes) and SWB in Chinese migrant older adults. Is migrant older adults’ SWB associated with the physical and social environment of neighbourhoods where they live? To the best of our knowledge, no studies for the China setting are available to date examining perceived neighbourhood environment and SWB in migrant older adults. 

To fill the knowledge gap, the present study aims to investigate the correlation between perceived neighbourhood environment and SWB amongst migrant older adults using canonical correlation analysis, which could inform the design of future interventions. Exploring the unique effects of neighbourhood attributes on migrant older adults’ SWB could be helpful to urban planners and public health officials in their efforts to build age-friendly neighbourhoods. The research will provide a reference and basis for individual behaviour decision making and community planning and governance.

## 2. Materials and Methods

### 2.1. Design

The present study employs a cross-sectional questionnaire survey conducted in Dongguan in South China, to determine the correlation between perceived neighbourhood environment and SWB amongst migrant older adults.

### 2.2. Subjects

This survey was performed amongst migrant older adults in Dongguan city between December 2018 and February 2019. The migrant older adults in this research were defined as any person aged not less than 60 years, those who had moved to Dongguan at least six months prior to the survey and were not listed in the household registration system of Dongguan. An eligible list of migrant older adults for the study was provided by the community committee. A multistage cluster sampling survey technique was used and 470 migrant older adults were invited to take part in the study (98.2% response rate). In the first stage, four districts were purposively selected out of 33 districts. In the second stage, 22 clusters were randomly selected from 26 communities with a probability proportional to the older adult’s density. In the third stage, within each cluster, migrant older adults were selected randomly. 

### 2.3. Measure

#### 2.3.1. Subjective Wellbeing (SWB)

SWB was assessed by the Memorial University of Newfoundland Scale of Happiness (MUNSH), which has been designed specifically for older adults and has high validity (Kaiser-Meyer-Olkin (KMO) of 0.703) and consistency (Cronbach’s alpha of 0.735) [40]. The MUNSH is a multiitem scale which has 24 items, assessing four dimensions: positive emotion (PA) [e.g., ‘*Generally satisfied with the way your life has turned out?*’], general positive experience (PE) [e.g., ‘*Are you satisfied with your life today?*’], negative emotion (NA) [e.g., ‘*Bitter about the way your life has turned out?*‘], and general negative experience (NE) [e.g., ‘*How much do you feel lonely?*’]. Numerous items on this scale cover specific content in the geriatric area with reference to age and time of life. Possible responses to each item are ‘yes’ (score 2 points), ‘I don’t know’ (1 point), and ‘no’ (0 points). The total SWB score was then calculated using the equation PA + PE − NA − NE. Total scores range between −24 to + 24 points, where higher scores indicate better SWB [40]. 

#### 2.3.2. Perceived Neighbourhood Environment (PNE)

The perceived neighbourhood environment in the present study consists of the physical and social environment attributes, namely ‘walkability of the neighbourhood’ and ‘social cohesion’. These two environment attributes were assessed by the related module of the Neighbourhood Scales developed by Mujahid [41]. The walkability of the neighbourhood was measured with seven items (The specific items are presented in Table 1), asking the participants if they believed that their neighbourhood offered opportunities and facilities for physical activities, has adequate green space and walkable places, and if they observed other people walking in their neighbourhood. The questionnaire uses a 5-point Likert Scale, ranging from 1 = strongly disagree to 5 = strongly agree with the statements. The Cronbach’s alpha of the original scale was 0.73 [20,41]. The total score ranges from 7–35. 

Social cohesion is comprised of four questions asking the respondent about their values such as interpersonal trust, and their relationship with their neighbours. This questionnaire also uses a 5-point Likert Scale, with responses ranging from 1 = strongly disagree to 5 = strongly agree with each statement. The Cronbach’s alpha of the original scale was 0.74 [20,41]. The total score ranges from 4–20.

#### 2.3.3. Individual Characteristics

The socio-demographic factors recorded were gender, age, living arrangements, health insurance, and pension status. Living arrangement was categorized as “living with child only”, “living with child and spouse”, “living with child and grandchild”, “living with child, grandchild and spouse”, and “living alone”. Health insurance and pension status were divided into a “have” group and a “haven’t” group. Self-rated health was divided into three ordinal categories: “good”, “fair”, and “poor”.

### 2.4. Data Collection

Nine research assistants (second-year postgraduates) and community staff were trained at a workshop. All interviewers were trained before the formal collection of data by an experienced researcher. The workshop included an introduction to the study and the methods and skills of conducting quantitative interviews. The questionnaires were tested in a pilot study. Face-to-face interviews using the structured questionnaire were conducted. All of the participants were interviewed at their homes using their local language by trained interviewers. Each interview took about 20–25 min. The supervisors checked the completion of the questionnaire during the fieldwork. If information was missing, the interviewer went back to obtain the missing information.

### 2.5. Data Analysis

SPSS V.26.0 software was used to process the data. A Pearson correlation analysis was used to analyse the correlations between the perceived neighbourhood environment variables (X1-X11) and the SWB dimensions (PA, NA, PE and NE). Canonical correlations between the perceived neighbourhood environment variables and the SWB dimensions were analysed after standardising the scores of each variable. 

Canonical correlation analysis is an approach that involves the application of structure coefficients as indices for the identification of important indicators. It is a multivariate statistical analysis method used to determine the correlation between two sets of variables using the correlation between the combined pairs of variables to reflect the overall correlation between the two sets of indicators [42]. This paper focuses on the correlation between the two sets of variables of neighbourhood environment and subjective wellbeing, so canonical correlation analysis was chosen.

Canonical redundancy reflects the percentage of variance explained by each canonical variable for each group of variables, If the canonical variables are well representative of the original variables, prediction can be made by canonical correlation. The magnitude of the redundancy analysis indicates the extent to which the pair of canonical variables can explain each other for another set of variances, and it will provide some useful information for further discussion of the relationship between many-to-many [42].

### 2.6. Ethical Considerations

Ethical approval was received from the Institutional Ethics Committee of the Ethics Review Committee of Guangdong Medical University, China (REC: PJ2018037) before the research was conducted. Privacy and data confidentiality were ensured. Voluntary participation and unconditional withdrawal were offered to all participants. A small gift was given as a thank you for their participation.

## 3. Results

The total sample consisted of 470 migrant older adults. Of those, 275 were female (58.5 percent) and 195 were male (41.5 percent). The mean age of the participants was 67.1 years (SD 5.5), with a minimum age of 60, and a maximum age of 87 years. Most participants had fair to good health (n = 424, 90.2 percent). Most of the migrant older adults lived with their families with an average of more than three members (n = 456, 97.0 percent). Approximately one-third of migrant participants lacked health insurance (n = 135, 28.7 percent) and had no pension (n = 166, 35.3 percent).

Table 2 illustrates the results of SWB variables and PNE variables. For SWB, the mean score (x ± s) of the total scores for SWB was 14.76 ± 8.31. For PNE, the mean score (x ± s) of walkability and social cohesion were 27.34 ± 5.15 and 15.69 ± 2.62, respectively.

The simple correlation analyses of PNE and SWB demonstrated that the correlations ranged between r = 0.276 and r = 0.423 for PA and PE, indicating there was a moderate level of correlation, whilst NA and NE were negatively correlated with X1-X3 and X8-X11, and it revealed a low level of correlation (Table 3).

The 11 variables X1–X11 of the above simple correlation analysis species were used as the X set, the scores of the SWB dimensions were used as the Y set for typical correlation analysis, and four common variables were obtained (Table 4).

The results revealed that within the four pairs of canonical variables, two pairs of canonical variables were statistically significant (r1 = 0.402, *p* < 0.0001 and r2 = 0.257, *p* < 0.05), demonstrating that there was a correlation between SWB and the PEN variables. The first pair of canonical variables contained 60.88 percent of the information. The first two pairs of typical variables cumulatively contributed to 82.87 percent of the information.

Table 5 reveals that in the first pair of canonical variables, residents with neighbourhood relations (X9), neighbourhood trust (X10), and similar values (X11) in social cohesion are positively correlated with PA (Y1) and PE (Y3). In the second pair of canonical variables, the walkability of X1, X2, X6, and X7 and the social cohesion of neighbours helping each other (X8) with PA (Y1), NA (Y2) in SWB, are closely correlated to each other.

Redundancy analysis (Table 6) demonstrated that amongst the first pair of canonical variables, *U*1 could explain 44.1 percent of the total variation in the X variable set and 7.1 percent in the Y variable set, whilst *V*1 could explain 53.0 percent of the total variation in the Y variable set and 8.6 percent in the X variable set. In the second pair of canonical variables, *U*2 could explain 3.8 percent of the total variation in the X variable set and 0.3 percent in the Y variable set, whilst *V*2 could explain 16.2 percent of the total variation in the Y variable set and 1.1 percent in the X variable set.

## 4. Discussion

This study explored the correlation between PNE and SWB among migrant older adults to understand the relative importance and level of the components of PNE and SWB. The results showed that migrant older adults with a high PNE have better PA and PE, which leads to a generally high SWB. This result is in line with previous studies, which suggested that higher PNE leads to higher SWB. Previous studies have confirmed that neighbourhood-built environments (e.g., walkability) and social environments affect older adults’ SWB (e.g., social cohesion) [8,11]. It is possible that this is due to the fact that older adults are more dependent on their neighbourhoods and that changes and adaptations in the neighbourhood environment have a greater impact on their lives [9,20].

Neighbourhood environments matter since they are socially structured and represent differential amenities, including access to physical resources, social support, and relationships [43]. Furthermore, the residential neighbourhood is the older adults’ predominant environmental context, particularly those who are retired or migrated with family [9,20]. Therefore, they likely spend more time increasingly with neighbours in the neighbourhood. Thus, this study provides evidence for the need to reinforce the neighbourhood environment for migrant older adults to improve their SWB by demonstrating a more comprehensive canonical correlation between the eleven elements of PNE and SWB.

The social environment attributes of PNE, their relationship with their neighbours, interpersonal trust, and sharing the same values are associated with positive emotions and wellbeing experiences, consistent with previous studies [8,20,43]. Older adults may be more affected by neighbourhood characteristics (e.g., social cohesion) than younger adults who have been reported in some studies as being more concerned about environmental pollution and neighbourhood beautification [44,45]. Social cohesion is an aspect of the neighbourhood’s social environment influencing individual health-related behaviours such as physical and recreational activities [46]. Social cohesion refers to the absence of potential social conflict and the presence of strong social bonds—usually measured by levels of trust and reciprocity norms [47].

Cohesive neighbourhoods may be better for reinforcing positive social norms for health behaviours, leading to quicker adoption of new residents since neighbours know and trust each other [11,12,22]. In addition, neighbours who trust one another are more likely to provide help and support in times of need, particularly for migrant older adults who face the dilemmas of losing geopolitical ties and have difficulty integrating into new cities [20,22]. Research has demonstrated that people may only trust those in the same in-group and may not participate in social activities outside their circle [48]. Therefore, migrant older adults who share the same values as neighbours are more likely to establish good relationships and trust each other, which leads to promoting their SWB.

The current study used SWB to examine the association of physical neighbourhood attributes and walkability. We found a link between SWB and walkable neighbourhoods characterized by opportunities and facilities for physical activities with other people walking or exercising in their community, positively associated with positive emotions and negatively associated with negative emotions. Previous studies showed neighbourhood walkability is related to leisure time physical activity among Chinese and U.S. older adults [49,50]. Walking is correlated with both improved physical and emotional health [51]. In addition, researchers found a link between walkable neighbourhood attributes that include land use diversity and well-connected transportation networks with more walking, less obesity, and lower coronary heart disease risk [52,53].

Migration, retirement, and other major life events tend to create anxiety, pessimism, depression, and other native emotions in migrant older adults [5]. However, a good walking environment provides migrant older adults with conditions for exercise and creates a platform for the older adults to communicate with their neighbours. Through walkable neighbourhoods, migrant older adults can avoid the “social isolation” phenomenon and “social insularity” caused by long-term absence from home [54]. It also helps them to improve their self-worth and maintain positive mental health while participating in social activities [54]. For example, Wiles (2012) found that a high-quality physical neighbourhood environment enhances wellbeing [55]. This effect is due to people having an innate emotional connection to their neighbourhood environment, and open spaces can increase social interaction.

This study is meaningful since our comprehensive analyses factored in the various elements of PNE to demonstrate the canonical correlation between PNE and SWB of migrant older adults. The study extends this prior research by focusing specifically on perceived neighbourhood environments—defined in this study as being the combined physical and social environments—of Chinese migrant older adults [9,11,12]. The statistical approach of using canonical correlation analysis is appropriate for identifying the associations between the two sets of variables of the physical and social environments, measured as walkability and social cohesion, with subjective wellbeing. Our study emphasized that positive physical and social environments are likely to contribute to the positive subjective wellbeing of the elderly. The findings could provide evidence to help governments design healthy ageing policies to improve the SWB at the community level. The findings also could potentially be expanded to other population groups. Positive physical and social environments are likely to contribute to positive subjective wellbeing beyond migrant older adults. However, this study has some limitations. First, we collected cross-sectional data based on self-reports. Thus, we cannot address the causality direction. Second, we conducted this study in only one city, which may not represent all migrant older adults in China. Therefore, future research should consider well-designed multicentre prospective studies of neighbour correlates of SWB. Third, the data collection instruments (i.e., focus on walkability) lacks accounts of other physical dimensions and amenities (i.e., health centres, banks, elderly activity centres, and parks) that support older people’s wellbeing and these could be highlighted as potential venues for further research. Finally, we did not assess the effect of changes in the socioeconomic status of the whole family on SWB and the participants’ ranges for time elapsed since migration which could affect migrant older adults’ SWB. Future research studies could explore this further.

There are some policy implications in this study’s findings. Our study suggests that physical and social attributes of neighbourhoods are strongly associated with migrant older adults’ SWB. Previous studies found that migrant older adults’ restricted access to social benefits and social relations was detrimental to their mental health [11,12]. Our findings confirm this point and further suggest that migrant older adults have a good walkable environment and social cohesion in neighbourhoods positively correlated with their subjective wellbeing. Therefore, the government should provide a more robust activity space for the neighbourhood and optimize the quality of life for older adults.

In addition, migrant older adults should be encouraged to participate in community activities to enrich their lives and improve their SWB. Finally, they could improve their wellbeing through inclusive community building. This approach requires breaking the closure and exclusion in the configuration of community power. Eliminating the identity segregation and social exclusion of residents in sharing community resources and promoting good neighbourliness among older adults with different identities and backgrounds will enhance migrant older adults’ sense of community cohesion and community belonging.

## 5. Conclusions

As residential migration becomes more common, the neighbourhood environment inevitably changes, and one needs to adapt to the new neighbourhood environment. This paper focused on older adults after residential migration. Initially, it explored the relationship between the new neighbourhood environment and SWB after migration, enriching the study of the relationship between neighbourhoods and SWB. However, we need further indepth analysis of how changes in the neighbourhood environment before and after residential migration affect older adults’ wellbeing and the critical role of other aspects of residential migration.

## Figures and Tables

**Table 1:** Specific items for walkability and social cohesion.

Module	Variable	Item	Response
Walkability	X1	My neighbourhood offers many opportunities to be physically active	seven items each scored on a 5-point scale from ‘1-strongly disagree’ to ‘5-strongly agree’
	X2	Local sports clubs and other facilities in my neighbourhood offer many opportunities to get exercise	
	X3	It is pleasant to walk in my neighbourhood	
	X4	The trees in my neighbourhood provide enough shade	
	X5	In my neighbourhood, it is easy to walk to places	
	X6	I often see other people walking in my neighbourhood	
	X7	I often see other people exercising (for example, jogging, bicycling, and playing sports) in my neighbourhood	
Social cohesion	X8	People around here are willing to help their neighbours	5 items each scored on a 5-point scale from ‘1-strongly disagree’ to ‘5-strongly agree’
	X9	People in my neighbourhood generally get along with each other	
	X10	People in my neighbourhood can be trusted	
	X11	People in my neighbourhood share the same values	

**Table 2:** Descriptive statistics of subjective wellbeing and perceived neighbourhood environment.

	Possible Range	Min	Max	Mean	SD
Subjective wellbeing					
PA	0–15	0	10	7.80	2.42
NA	0–15	0	10	1.80	2.44
PE	0–21	0	14	10.66	3.05
NE	0–21	0	14	1.89	2.67
Perceived neighbourhood environment					
X1	1–5	1	5	3.65	1.06
X2	1–5	1	5	3.65	1.03
X3	1–5	1	5	4.00	0.83
X4	1–5	1	5	3.96	0.90
X5	1–5	1	5	4.05	0.81
X6	1–5	1	5	4.07	0.80
X7	1–5	1	5	3.95	0.89
X8	1–5	1	5	3.96	0.77
X9	1–5	1	5	4.07	0.76
X10	1–5	1	5	3.95	0.77
X11	1–5	1	5	3.70	0.80

**Table 3:** Simple correlation analysis between subjective wellbeing dimensions scores and scores of perceived neighbourhood environment items.

Variable	PA	NA	PE	NE
X1	0.300 **	−0.144 *	0.341 **	−0.130 *
X2	0.325 **	−0.096 *	0.347 **	−0.107 *
X3	0.348 **	−0.095 *	0.332 **	−0.124 *
X4	0.302 **	−0.061	0.337 **	−0.072
X5	0.304 **	−0.081	0.312 **	−0.084
X6	0.297 **	−0.069	0.293 **	−0.016
X7	0.310 **	−0.162 **	0.333 **	−0.071
X8	0.276 **	−0.181 **	0.350 **	−0.175 **
X9	0.348 **	−0.147 **	0.360 **	−0.139 *
X10	0.380 **	−0.206 **	0.387 **	−0.213 **
X11	0.423 **	−0.162 **	0.368 **	−0.175 **

* Means that the correlation is significant when the confidence level (two-sided) is 0.05. ** Means that the correlation is significant when the confidence level (two-sided) is 0.01.

**Table 4:** Canonical correlation analysis of the scores of subjective wellbeing dimensions with perceived neighbourhood environment variables X1–X11.

n	Correlation Coefficient	Eigenvalue	Cumulative Proportion %	Wilk’s	Approximate F Value	df	*p* Value
1	0.402	0.193	60.88	0.743	3.199	44	<0.0001
2	0.257	0.070	82.87	0.886	1.879	30	0.003
3	0.191	0.038	94.95	0.948	1.363	18	0.142
4	0.125	0.016	100.00	0.984	0.905	8	0.512

**Table 5:** The correlation coefficients between the scores of the subjective wellbeing dimensions and perceived neighbourhood environment variables.

PNE Variables			Scores of SWB Dimensions			
Variable	*U*1	*U*2	SWB Dimensions	Variable	*V*1	*V*2
X1	−0.141	0.807	PA	Y1	−0.568	−0.968
X2	−0.145	−0.678	NA	Y2	0.159	−0.965
X3	−0.207	−0.307	PE	Y3	−0.515	0.491
X4	−0.163	−0.174	NE	Y4	−0.082	0.085
X5	0.145	0.029				
X6	0.211	−0.615				
X7	−0.176	0.968				
X8	0.140	0.745				
X9	−0.245	−0.224				
X10	−0.295	0.150				
X11	−0.414	0.565				

**Table 6:** Redundancy analysis for canonical correlation analysis.

	X Set × Self	X Set × Y Set	Y Set × Self	Y Set × X Set
1	0.441	0.071	0.530	0.086
2	0.038	0.003	0.162	0.011

## Data Availability

Data are available upon request from the corresponding author.

## References

[1] Layard R. (2010). Measuring subjective well-being. Science.

[2] Oswald A.J., Wu S. (2010). Objective confirmation of subjective measures of human well-being: Evidence from the USA. Science.

[3] Diener E. (2000). Subjective well-being. The science of Happiness and a proposal for a national index. Am. Psychol..

[4] Diener E., Sapyta J.J., Suh E. (1998). Subjective well-being is essential to well-being. Psychol. Inq..

[5] Li S., Huang Z.Y. (2018). Subjective health conditions and the influencing factors of the elderly migrating population in Chinese megacities. J. Shenzhen Univ..

[6] Liu Y., Ingviya T., Sangthong R., Wan C. (2021). Subjective well-being and quality of life of rural-to-urban migrant and local adults in Dongguan, China. Soc. Behav. Personal. Int. J..

[7] Lovejoy K., Handy S., Mokhtarian P. (2010). Neighborhood satisfaction in suburban versus traditional environments: An evaluation of contributing characteristics in eight California neighborhoods. Landsc. Urban Plan..

[8] Liu Y., Sangthong R., Ingviya T., Wan C. (2022). Determinants of subjective well-being among migrant and local elderly in China: A cross-sectional study. BMJ Open.

[9] Liu Y., Jia L., Xiao J., Chen Q., Gan Q., Huang J., Zhu X., Zhang C., Wan C. (2022). The subjective well-being of elderly migrants in Dongguan: The role of residential environment. Trop. Med. Infect. Dis..

[10] Choi Y., Kwon Y.H., Kim J. (2018). The effect of the social networks of the elderly on housing choice in Korea. Habitat Int..

[11] Liu Y.Q., Zhang F.Z., Wu F.L., Liu Y., Li Z.G. (2017). The subjective well-being of migrants in Guangzhou, China: The impacts of the social and physical environment. Cities.

[12] Liu Y., Sangthong R., Ingviya T., Wan C. (2020). Subjective well-being of older adult internal migrants in China: The role of social environment. Soc. Behav. Personal. Int. J..

[13] Hooghe M., Vanhoutte B. (2011). Subjective well-being and social capital in Belgian communities. The impact of community characteristics on subjective well-being indicators in Belgium. Soc. Indic. Res..

[14] Chen Y., Ning Y. (2015). The impact of community environment on residents’ subjective well-being. Urban Issues.

[15] Kim T.K., Horner M.W., Marans R.W. (2005). Life cycle and environmental factors in selecting residential and job locations. Hous. Stud..

[16] Galster G., Ham M., Manley D., Bailey N. (2012). The mechanism(s) of neighbourhood effects: Theory, Evidence and Policy Implications. Neighbourhood Effects Research: New Perspectives.

[17] Jivraj S., Norman P., Nicholas O., Murray E.T. (2019). Are there sensitive neighbourhood effect periods during the life course on midlife health and wellbeing. Health Place.

[18] Clark W.A.V., Withers S.D. (2007). Family migration and mobility sequences in the United States: Spatial mobility in the context of the life course. Demogr. Res..

[19] Parra D.C., Gomez L.F., Sarmiento O.L., Buchner D., Brownson R., Schimd T., Gomez V., Lobelo F. (2010). Perceived and objective neighborhood environment attributes and health related quality of life among the elderly in Bogota, Colombia. Soc. Sci. Med..

[20] Gao J., Fu H., Li J., Jia Y. (2015). Association between social and built environments and leisure-time physical activity among Chinese older adults—A multilevel analysis. BMC Public Health.

[21] Wang F.L., Wang D.C. (2020). Changes in residential satisfaction after home relocation: A longitudinal study in Beijing, China. Urban Stud..

[22] Liu Y., Sangthong R., Ingviya T., Wan C. (2019). Nothing like living with a family: A qualitative study of subjective well-being and its determinants among migrant and local elderly in Dongguan, China. Int. J. Environ. Res. Public Health.

[23] Schwane T., Wang D.C. (2014). Well-being, context, and every-day activities in space and time. Ann. Assoc. Am. Geogr..

[24] Nakazato N., Schimmack U., Oishi S. (2011). Effect of changes in living conditions on well-being: A prospective top-down bottom-up model. Soc. Indic. Res..

[25] Gerber P., Ma T.Y., Klein O., Schiebel J., Carpentier S. (2017). Cross-border residential mobility, quality of life and modal shift: A Luxembourg case study. Transp. Res. Part A Policy Pract..

[26] Bucx F., Wel F., Knijn T., Hagendoorn L. (2008). Intergenerational contact and the life course status of young adult children. J. Marriage Fam..

[27] Mulder C.H., Meer M.J. (2009). Geographical distances and support from family members. Popul. Space Place.

[28] Leopold T., Geissler F., Pink S. (2012). How far do children move? Spatial distances after leaving the parental home. Soc. Sci. Res..

[29] Schutt R.K., Goldfinger S.M., Penk W.E. (1997). Satisfaction with residence and with life: When homeless mentally ill persons are housed. Eval. Program Plan..

[30] Nowok B., Findlay A., McCollum D. (2018). Linking residential relocation desires and behaviour with life domain satisfaction. Urban Stud..

[31] Lundholm E., Malmberg G. (2006). Gains and losses, outcomes of interregional migration in the five Nordic countries. Geogr. Ann. Ser. B Hum. Geogr..

[32] Liu Y., Xu W. (2015). Destination choices of permanent and temporary migrants in China, 1985–2005. Popul. Space Place.

[33] Zhang J. (2014). Enhancing Quality of Life: The Social Support of Elderly Chinese Migrants in New Zealand. Ph.D. Thesis.

[34] Shen Y., Yeatts D.E. (2013). Social support and life satisfaction among older adults in China: Family-based support versus community-based support. Int. J. Aging Hum. Dev..

[35] Day R. (2008). Local environments and older people’s health: Dimensions from a comparative qualitative study in Scotland. Health Place.

[36] Su L., Zhou S., Zhang X., Zhang L. (2019). The Impact of Community Environment on Residents’ Subjective Well-being: The Role of the Time Dimension. Res. Urban Dev..

[37] Bustamante G., Guzman V., Kobayashi L.C., Finlay J. (2022). Mental health and well-being in times of COVID-19: A mixed-methods study of the role of neighborhood parks, outdoor spaces, and nature among US older adults. Health Place.

[38] Finlay J., Franke T., McKay H., Sims-Gould J. (2015). Therapeutic landscapes and wellbeing in later life: Impacts of blue and green spaces for older adults. Health Place.

[39] Ambrey C. (2012). Public Green Space and Life Satisfaction in Urban Australia. Urban Stud..

[40] Kozma A., Stones M.J. (1980). The measurement of Happiness: Development of the Memorial University of Newfoundland scale of Happiness (MUNSH). J. Gerontol..

[41] Mujahid M.S., Diez Roux A.V., Morenoff J.D. (2007). Assessing the measurement properties of neighborhood scales: From psychometrics to ecometrics. Am. J. Epidemiol..

[42] Ronald P., Cody Jeffrey K. (2005). Applied Statistics and SAS Programming Language.

[43] Liu Y., Dijst M., Geertman S. (2017). The subjective well-being of older adults in Shanghai: The role of residential environment and individual resources. Urban Stud..

[44] Elliott J., Gale C.R., Parsons S. (2014). Neighbourhood cohesion and mental wellbeing among older adults: A mixed methods approach. Soc. Sci. Med..

[45] Mulliner E., Riley M., Maliene V. (2020). Older people’s preferences for housing and environment characteristics. Sustainability.

[46] Cradock A.L., Kawachi I., Colditz G.A., Gortmaker S.L., Buka S.L. (2009). Neighborhood social cohesion and youth participation in physical activity in Chicago. Soc. Sci. Med..

[47] Berkman L.F., Kawachi I. (2000). Social Epidemiology.

[48] Allik J., Realo A. (2004). Individualism-collectivism and social capital. J. Cross-Cult. Psychol..

[49] Carlson J.A., Sallis J.F., Conway T.L., Saelens B.E., Frank L.D., Kerr J., Cain K.L., King A.C. (2012). Interactions between psychosocial and built environment factors in explaining older adults’ physical activity. Prev. Med..

[50] Cerin E., Lee K.Y., Barnett A., Sit C.H., Cheung M.C., Chan W.M. (2013). Objectively-measured neighborhood environments and leisure-time physical activity in Chinese urban elders. Prev. Med..

[51] Suarez-Balcazar Y., Early A.R., Garcia C., Balcazar D., Arias D.L., Morales M. (2020). Walkability Safety and Walkability Participation: A Health Concern. Health Educ. Behav..

[52] Lovasi G.S., Grady S., Rundle A. (2012). Steps forward: Review and recommendations for research on walkability, physical activity and cardiovascular health. Public Health Rev..

[53] King A.C., Sallis J.F., Frank L.D., Saelens B.E., Cain K., Conway T.L., Chapman J.E., Ahn D.K., Kerr J. (2011). Aging in neighborhoods differing in walkability and income: Associations with physical activity and obesity in older adults. Soc. Sci. Med..

[54] Roh S., Jang Y., Chiriboga D.A., Kwag K.H., Cho S., Bernstein K. (2011). Perceived neighborhood environment affecting physical and mental health: A study with Korean American older adults in New York city. J. Immigr. Minor. Health.

[55] Wiles J.L., Leibing A., Guberman N. (2012). The meaning of “aging in place” to older people. Gerontologist.

